# Expression profile of G-protein βγ subunit gene transcripts in the mouse olfactory sensory epithelia

**DOI:** 10.3389/fncel.2013.00084

**Published:** 2013-06-04

**Authors:** Aaron Sathyanesan, Adrian A. Feijoo, Saloni T. Mehta, Akua F. Nimarko, Weihong Lin

**Affiliations:** Department of Biological Sciences, University of Maryland Baltimore County, BaltimoreMD, USA

**Keywords:** olfactory sensory neurons, vomeronasal, postnatal development, sensory transduction, TRPM5, phospholipase C

## Abstract

Heterotrimeric G-proteins mediate a variety of cellular functions, including signal transduction in sensory neurons of the olfactory system. Whereas the Gα subunits in these neurons are well characterized, the gene transcript expression profile of Gβγ subunits is largely missing. Here we report our comprehensive expression analysis to identify Gβ and Gγ subunit gene transcripts in the mouse main olfactory epithelium (MOE) and the vomeronasal organ (VNO). Our reverse transcriptase PCR (RT-PCR) and realtime qPCR analyses of all known Gβ (β_1,2,3,4,5_) and Gγ (γ_1,2,2*t*,3,4,5,7,8,10,11,12,13_) subunits indicate presence of multiple Gβ and Gγ subunit gene transcripts in the MOE and the VNO at various expression levels. These results are supported by our RNA *in situ* hybridization (RISH) experiments, which reveal the expression patterns of two Gβ subunits and four Gγ subunits in the MOE as well as one Gβ and four Gγ subunits in the VNO. Using double-probe fluorescence RISH and line intensity scan analysis of the RISH signals of two dominant Gβγ subunits, we show that Gγ_13_ is expressed in mature olfactory sensory neurons (OSNs), while Gβ_1_ is present in both mature and immature OSNs. Interestingly, we also found Gβ_1_ to be the dominant Gβ subunit in the VNO and present throughout the sensory epithelium. In contrast, we found diverse expression of Gγ subunit gene transcripts with Gγ_2_, Gγ_3_, and Gγ_13_ in the Gα_i2_-expressing neuronal population, while Gγ_8_ is expressed in both layers. Further, we determined the expression of these Gβγ gene transcripts in three post-natal developmental stages (p0, 7, and 14) and found their cell-type specific expression remains largely unchanged, except the transient expression of Gγ_2_ in a single basal layer of cells in the MOE during P7 and P14. Taken together, our comprehensive expression analyses reveal cell-type specific gene expression of multiple Gβ and Gγ in sensory neurons of the olfactory system.

## Introduction

Sensory neurons in peripheral olfactory systems employ G-protein coupled signaling pathways to transduce chemical stimuli. G-proteins are heterotrimeric, composed of three different subunits—α, β, and γ. To date, gene transcripts encoding proteins of 20 α subunits, 5 β subunits and 12 γ subunits have been identified (Malbon, 2005; Dupre et al., 2009) in mammals. Out of the 20 known Gα subunits, Gα_olf_, Gα_s_, Gα_o_, and Gα_i2_ subunit proteins are known to be expressed in the main olfactory system (Pace and Lancet, 1986; Jones and Reed, 1989; Wekesa and Anholt, 1999) and Gα_o_ and Gα_i2_ subunit proteins are known to be expressed in the vomeronasal sensory organ (VNO) (Berghard and Buck, 1996).

Gα subunit gene expression in the MOE and VNO is regulated developmentally, and also in a cell-type specific context (Halpern et al., 1995; Sullivan et al., 1995). Post-natally, mature olfactory sensory neurons (OSNs) primarily express the stimulatory Gα_olf_ subunit and immature OSNs express Gα_s_ proteins (Menco et al., 1994) while Gα_o_ and Gα_i2_ proteins are found in the axons of OSNs (Wekesa and Anholt, 1999; Luo et al., 2002). A differential expression pattern of α subunits is also seen in vomeronasal sensory neurons (VSNs), with apical VSNs expressing Gα_i2_ and basal VSNs expressing Gα_o_ proteins (Halpern et al., 1995; Berghard and Buck, 1996). Mice with genetic ablation of Gα_olf_ and Gα_o_ from the MOE and the VNO respectively, exhibit severe deficits in olfactory-associated behaviors (Belluscio et al., 1998; Chamero et al., 2011). These studies provide strong evidence for the specific roles of each G-protein α subunit, however, whether β and γ subunits can be activated in the sensory neurons of these knockout mice remains to be determined.

It has become increasingly clear in other systems, such as the cardiovascular and taste systems that following receptor activation, the Gβγ dimer dissociates from the Gα subunit and modulates a set of downstream components that are different from those activated by Gα (Dingus et al., 2005; Smrcka, 2008). For example, in mammalian taste receptor cells, upon tastant binding to the taste receptor, the Gβγ subunit dissociates from the α subunit gustducin (Gα_gust_) and activates phospholipase C β_2_ (PLC β_2_) (Huang et al., 1999) which in turn activates the DAG/IP_3_ pathway and transient receptor potential channel M5 (TRPM5) (Oike et al., 2006; Zhang et al., 2007). Gene and protein expression data for solitary chemosensory cells (SCCs) in the gut and the respiratory tract as well as for the putative glucose-sensing hypothalamic neurons, which express taste receptor T1R1, T1R2, and T1R3, also indicates the presence of a similar Gβγ-mediated activation of the PLC pathway (Bezencon et al., 2007; Lin et al., 2008; Ren et al., 2009; Krasteva et al., 2011). In invertebrates, Gγ_1_ has been implicated in the detection of sugar by *Drosophila* taste neurons, and GPC-1, one of the Gγ subunit homologs in *C.elegans*, has been implicated in taste (Jansen et al., 2002) and olfactory adaptation (Yamada et al., 2009). These accumulating evidences clearly demonstrate the important role of Gβγ subunits in chemical sensing.

In the mouse MOE, canonical mature olfactory sensory neurons (mOSNs) employ a G-protein-mediated, cAMP signal transduction cascade (Bakalyar and Reed, 1990; Munger et al., 2009). However, data regarding the expression of β and γ subunits of G-proteins in mOSNs and the epithelium, in general, is scarce and scattered. Previously, Ryba and Tirindelli (1995) reported the expression of Gγ_8_ gene transcript in cell bodies of immature OSNs (iOSNs) using RNA in situ hybridization (RISH). In addition, Gγ_13_ protein is known to express strongly in cilia and cell bodies of mOSNs in the MOE, including a subset of OSNs that express TRPM5, and plays an important role in olfaction (Kulaga et al., 2004; Lin et al., 2007; Li et al., 2013). More recently, Kerr et al. (2008) reported that Gβ_1_ was the dominant Gβ subunit in the MOE. Boto et al. (2010) used RT-PCR to demonstrate the presence of 3 Gβ and 2 Gγ subunit transcripts in the olfactory organs of *Drosophila*. Yet, to date, there is no comprehensive analysis regarding Gβγ subunit gene expression profiles in the MOE and VNO and their developmental regulation in mammalian olfactory epithelia.

We sought to establish a cell-type specific expression profile of Gβ and Gγ subunit gene transcripts in the mouse MOE and VNO. We extracted total RNA from MOE and VNO tissue and conducted *in vitro* transcription and reverse-transcriptase PCR (RT-PCR) analysis for all known Gβ and Gγ subunits. We also conducted realtime quantitative PCR (qPCR) to determine quantitatively the expression levels of the Gβ and Gγ subunits. In addition, we conducted RISH analysis to determine their cell-type specific expression based on the PCR results. Further, we investigated postnatal developmental changes in the gene expression pattern of various subunits in P0, P7, and P14 MOE and VNO. Our results reveal cell-type specific expression of Gβ and Gγ subunit gene transcripts in the MOE and VNO, and provide a systematic analysis of the post-natal developmental profile of these subunits in peripheral olfactory epithelia.

## Materials and methods

### Animals

Wildtype C57BL/6 mice of both sexes at different ages including post-natal day 0, 7, 14, and adult (2–4 months) were used for experiments. All animal care and procedures were approved by the Animal Care and Use Committee of University of Maryland, Baltimore County.

### Reverse transcriptase PCR (RT-PCR)

#### Primer design

Primers were designed to amplify a partial sequence from the 3′UTR region of each of the β and γ mRNA found in mice, such that the expected amplicons would have least homology compared to another member within the β and γ subfamilies. Primers for RT-PCR were designed using Vector NTI software (Life technologies, Carlsbad, CA) and custom-made from IDT (Coralville, IA). Primer sequences and expected sizes of amplicons are listed in Table 1.

**Table 1:** **RT-PCR oligonucleotide primer sequences for Gβγ subunits**.

**Gene symbol**	**NCBI GI no**.	**RT-PCR primer sequence (5′ = Forward; 3′ = Reverse)**	**Expected amplicon size (bp)**
**G**β **SUBUNITS**			
*Gnb1* (β_1_)	111186467	5′: CCTGGACATGGCAAAGAGAATACAG	200
		3′: CCTCATGTCAAACTGCTTTATTACATC	
*Gnb2* (β_2_)	141803173	5′: TGCCCATGCCCACACTACAGG	335
		3′: CAGAGTTGGAAGTGGTTCCTTTAT	
*Gnb3* (β_3_)	20502975	5′: GGAGGCTAGAGGAAGAGGTGGGAA	367
		3′: GGGAAGGAAGCCAGGAGACTAGG	
*Gnb4* (β_4_)	145301555	5′: TTCTGTTCTCCAATGATACCTGG	236
		3′: ATGAATACCCTGGCCTTTGACC	
*Gnb5* (β_5_)	158518005	5′: CTCGTGTAGATATGACTTCTCCATGAG	292
		3′: GAAGACAGACTAGATCCAAGGAAAC	
**G**γ **SUBUNITS**			
*Gng1* (γ_1_)	142366390	5′: GGAAGTGACACTGGAGAGAATGAT	545
		3′: CCAGCCTGGTCTACAGAGTG	
*Gng2* (γ_2_)	84490416	5′: GCCAGCAACAACACCGCCAG	256
		3′: ATGTCCCAGGAGCCCCAACAC	
*Gngt2* (γ_2*t*_)	113461991	5′: CCCACCCTCACCACCATCAC	115
		3′: TTTTCTCAGCATCTTTATTT	
*Gng3* (γ_3_)	84579907	5′: CCCCCGTTAACAGCACTATG	236
		3′: TCAGAGGAGGTCCACCGCTCT	
*Gng4* (γ_4_)	31542900	5′: AAGGAAGGCATGTCTAATAACAGCAC	260
		3′: ACAGCAGGAAAGGGCCCG	
*Gng5* (γ_5_)	84579905	5′: TTCTTCTAGCGTCGCCGCCA	239
		3′: GGTTCATGAAAAGTGGTTTGAGA	
*Gng7* (γ_7_)	84579914	5′: GCGCATTGAAGCTGGA	189
		3′: GAGATGGGGAAGAGAGAGAGA	
*Gng8* (γ_8_)	84579910	5′: TGGCCAAGATTGCTGAGG	243
		3′: GGATTCATACTTCTGCGGGGG	
*Gng10* (γ_10_)	84490417	5′: TTCCGGGGCCAGCGTGA	221
		3′: GCGAGCTTCTTCCCAGTCT	
*Gng11* (γ_1_1)	40254516	5′: CGCAAAGAAGTCAAGTTGCAG	177
		3′: ATTTCCCTCCCCCAGAGTT	
*Gng12* (γ_12_)	142363813	5′: TCCAGCAAGACGGCAAGC	267
		3′: CAGGTTGCTGCTGTGGTTTGCG	
*Gng13* (γ_13_)	157951662	5′: ATGGAGGAGTGGGATGTGC	204
		3′: TCATAGGATGGTGCACTTGG	

#### RNA extraction, cDNA synthesis and gel electrophoresis

Total RNA was extracted using Nucleospin RNA II kit (Macherey-Nagel, Dueren, Germany) from homogenized samples of freshly dissected tissue, peeled from the olfactory turbinates and vomeronasal sensory epithelium. Five hundred nanogram (ng) of total RNA template was used for cDNA synthesis using iScript cDNA Synthesis Kit (Bio-Rad, Hercules, CA) and 1 μ l of synthesized cDNA was used as starting template for PCR using specific primers against each of the five β and twelve γ subunit gene transcripts. For control of genomic DNA contamination, we omitted the reverse transcriptase (RT) in the cDNA synthesis step, which resulted in no visible PCR products (data not shown). The PCR products were run on a 2% agarose gel and viewed using a UV transilluminator. Gel images were captured using MultiDoc-It™ Imaging System (UVP, Upland, CA).

### Realtime quantitative PCR (QPCR)

For realtime PCR, reverse primer sequences for each of the β and γ are furnished in Table 2. The same forward primer used for regular reverse transcriptase PCR, was used in the qPCR reaction. Maxima SYBR Green/ROX qPCR Master Mix (Thermo Scientific, Waltham, MA) was used to perform qPCR in an iCycler IQ Real-Time Detection system (Bio-Rad). The qPCR data were analyzed using the relative quantification method (2^−Δ ΔCt^) described by Livak and Schmittgen (2001) with *Gapdh* gene expression serving as the internal reference following the manufacturer's instruction manual.

**Table 2:** **qPCR reverse oligonucleotide primer sequences for Gβγ subunits**.

**Gene symbol**	**Reverse (3′) primer for qPCR**
**G**β **subunits**	
*Gnb1* (β_1_)	AGGTGAAAAGGGTACAGGGTGCAG
*Gnb2* (β_2_)	GGTATGGGAGCAGGACCGGAGG
*Gnb3* (β_3_)	GACTGACCCTAAGGAGACGGGGC
*Gnb4* (β_4_)	GCAAATAGAAGGTAGAGGGTTGG
*Gnb5* (β_5_)	ACACTGGACGGGGTGAGTGAGTG
**Gγ SUBUNITS**	
*Gng1* (γ_1_)	AGAGGGTCTTCTCCAGACG
*Gng2* (γ_2_)	TGGTCCTTAGGTATCCCAGTACA
*Gngt2*(γ_2*t*_)	TGGTCCTTAGGTATCCCAGTACA
*Gng3* (γ_3_)	GCCTTGGACACCTTTATCCGGCA
*Gng4* (γ_4_)	TCACCCTGTCCATGCAGGCTTC
*Gng5* (γ_5_)	ACCTTCACGCGGTTGAGCCC
*Gng7* (γ_7_)	AGCAAGGGATCGTTGCGGGC
*Gng8* (γ_8_)	GCTGCTGCCTGCGACACCTT
*Gng10* (γ_10_)	AGCTCTGCAGCTGCCTGGGA
*Gng11* (γ_11_)	ATTTCCCTCCCCCAGAGTT
*Gng12* (γ_12_)	GCTTCCAATCTCAGCTGCTGCAC
*Gng13* (γ_13_)	GCTCGGGGATGGTCTTGGACG

### RNA *in situ* hybridization (RISH)

#### RNase-free conditions

All solutions used as a part of RISH, until the post-hybridization washes, were made using water treated with diethyl pyrocarbonate (DEPC; Sigma-Aldrich Co., St. Louis, MO) to a final concentration of 0.1%, stirred overnight, and autoclaved to remove excess DEPC. In general, RNase-free conditions were maintained (e.g., using RNase Zap [Sigma] for tabletops) for all RNA-related work including riboprobe synthesis.

#### Tissue preparation

The procedure for tissue preparation was modified from our previous studies (Ogura et al., 2010, 2011). Briefly, mice (P7, P14, and adult) were anesthetized using Avertin (250 μg/g body weight), transcardially perfused with 0.1 M phosphate buffer (PB), followed by 4% paraformaldehyde (PFA) buffered in PB. P0 mice were anesthetized by hypothermia and then perfused. The nose was harvested, post-fixed for 2 h, and then further fixed overnight at 4°C in 4% PFA containing 25% sucrose. After overnight fixation, the nose was deboned and embedded with optimal cutting temperature (OCT) compound (Sakura Finetek, USA Inc., Torrance CA). The embedded nose was cut into 14 μm-thick coronal sections using a cryostat (Microm international, Walldorf, Germany) and mounted onto Superfrost plus slides (Fisher Science, Pittsburgh, PA). Slides were stored in a −80°C freezer till use.

#### Riboprobe synthesis

Riboprobes were synthesized using a protocol adapted and modified from Ishii et al. (2004). Briefly, RT-PCR amplicons of β and γ subunit gene transcripts detected in the MOE and the VNO were cloned into pGEMT-Easy vector (Promega, Fitchburg, WI). The plasmid was then linearized using restriction enzymes *Nde*I or *Sph*I (New England Biolabs, Ipswich, MA) to make antisense and sense riboprobes depending on orientation of insert in PGEMT-Easy (i.e., if the insert had +/+ orientation, then *Sph*I-linearized plasmid was used to make antisense riboprobe and *Nde*I-linearized plasmid was used to make sense riboprobe, else, if the insert orientation was +/−, then the method was switched). The linearized plasmids were then used as templates in an *in vitro* transcription reaction, containing either digoxygenin-UTP (DIG) or fluorescein-UTP (FLU) to generate required antisense (+/+; *Sph*I; Sp6 RNA polymerase [Thermo Scientific]) and sense riboprobes (+/+; *Nde*I; T7 RNA polymerase [Thermo Scientific]). The riboprobes were purified, dissolved in DEPC-treated water and stored at −20°C till use.

#### Probe hybridization

The protocol described in Ishii et al. (2004) was modified for probe hybridization and post-hybridization washes (Ishii et al., 2004). Briefly, following an initial fixation step for 15 min in 4% PFA, slides containing tissue sections were washed in PBS and subjected to 30 min of hydrogen peroxide (0.1% in PBS) treatment to quench endogenous peroxidases. Sections were then incubated in proteinase K solution (10 μg/ml in TE) for 7 min, subsequently fixed again in 4% PFA for 10 min, acetylated for 10 min followed by sequential dehydration (90 s each) with increasing concentrations of ethanol. After equilibration in hybridization solution, riboprobes dissolved in hybridization solution were applied to each slide (0.2 μg/ml of stock probe; 200 μl/slide). A piece of parafilm was placed over slides and placed in a moist hybridization chamber, which was transferred into a preheated 65°C incubation chamber and allowed to hybridize overnight (12–16 h).

#### Probe detection

After post-hybridization washes, slides were equilibrated in 10% Normal donkey serum (in TN buffer). Anti-digoxygenin antibody (anti-digoxygenin-AP, Fab fragment from sheep, 1:1000; Roche, Indianapolis, IN) alone for single-label RISH, and in combination with anti-fluorescein antibody (anti-fluorescein-POD Fab fragment from sheep, 1:150; Roche) was applied to each slide and incubated at 4°C overnight (~12 h). Post-incubation, slides were washed and equilibrated with a magnesium-based buffer (0.1 M Tris pH 9.5, 0.1 M NaCl, 50 mM MgCl_2_).

For single-label colorimetric RISH, 250 μl nitro blue tetrazolium/5-bromo-4-chloro-3′-indolyl phosphate (NBT/BCIP; 1 tablet/10 ml water; Sigma-Aldrich) was applied to each slide and incubated in the dark till control antisense slides developed a purple coloration in the appropriate regions of the tissue sections. Care was taken not to overexpose the slides by monitoring slides applied with sense riboprobe. Slides were washed in PBS and then mounted with Fluoromount-G (Southern Biotech, Birmingham, AL) and covered with a coverslip.

For double-label fluorescence RISH, the anti-fluorescein probe signal was first amplified using tyramide signal amplification system (TSA Biotin System, PerkinElmer, Waltham, MA). Fluorescein antibody was then detected by applying 200 μl streptavidin-Alexa 488 (1:300, Life technologies, Grand Island, NY) per slide and incubated for 30 min in a moisture chamber. Slides were then washed and 600 ml of HNPP/Fast Red solution (Roche) at working concentration was applied to each slide and incubated in the dark for 30 min. Slides were then washed and mounted using Fluoromount and covered with a coverslip.

### Image acquisition

For single-label colorimetric RISH, images (2048 × 2048 pixels) were taken using 4× and 20× objective lens of an Olympus BX 41 compound microscope equipped with a Retiga 4000 R digital camera (QImaging, British Columbia, Canada). Color images were captured using bright light filtered through an accessory RGB color filter (CRI Micro^*^Color 2, QImaging). Image-Pro Plus 6.2 software (Media cybernetics, Bethesda, MD) was used for image capturing. For double-label fluorescence RISH, confocal fluorescence images were taken using an Olympus BX 61 epifluorescence microscope equipped with a spinning disc confocal unit and Hamamatsu Orca-AG digital camera (Hamamatsu Photonics, Japan). Slidebook 4.0 software (3i, Denver, CO) was used for confocal image capturing and processing.

### Line intensity scan analysis

MOE coronal section singly labeled with antisense probes against GAP43, Gβ_1_, and Gγ_13_ gene transcripts, respectively were used for the analysis and RISH signal in images was quantified using a modified version of the line intensity scan protocol described in Sathyanesan et al. (2012). Briefly, RISH images (32-bit RGB color, 2048 × 2048 pixels, pixel resolution of 0.372 μm/pixel with 20× objective lens) of tissue-sections of posterior and anterior MOE (2 images per antisense probe per mouse) were opened in NIH ImageJ (v 1.47i) and converted to an inverted image using the “invert” tool (*Edit*→*Invert)*. Two individual line scans (*Analyze*→*Plot Profile)*, oriented perpendicular to the tangent to the curvature of the basal lamina were obtained from each image (see Figure 3A insets for representative line scan regions, 56, 58, and 58 μm epithelial length line scans in Gβ_1_, GAP43, and Gγ_13_ image insets respectively). In images of anterior MOE, two line scans were performed. One was acquired from the septal region and the other from the lateral edge of the dorsal arch. In images of posterior MOE, one line scan was acquired from the septal region and the other from the endoturbinate. Scan profiles were imported into OriginPro 8.6 (Student version) statistical software suite. Epithelial lengths (X axis) as well as pixel intensity values (Y axis) in the profiles were normalized to the highest respective value in each profile. Average line scan profiles were obtained for each animal (from 2 scans/image × 2 images/animal = 4 scans per animal) using the “Average Multiple Curves” tool (*Analysis*→*Mathematics*→*Average Multiple Curves*→*Open Dialog)* over the full X range, keeping the number of points constant at 500 interpolation points per averaged trace, using linear interpolation. 500-datapoint averages were then normalized within an animal and averaged over three animals, keeping the number of points constant at 100 for the final averaged trace and reporting SEM per point. Area integrals between specified points were calculated using the “Integrate” gadget in OriginPro 8.6 (*Gadgets*→*Integrate)*.

### Statistical analysis

Kolmogorov-Smirnov test was used to determine the significant difference between Gβ_1_ distribution and Gγ_13_ distribution within the GAP43 label (x1 = 0.63 to x2 = 0.93). For qPCR data analysis, One-way ANOVA followed by Tukey's *post-hoc* analysis was performed to determine statistically significant differences in the expression among Gβ and Gγ subunit gene transcripts. All statistical analyses were carried out using OriginPro 8.6 software.

## Results

### RT-PCR analysis of Gβ and Gγ gene transcript expression in sensory epithelia of the peripheral olfactory system

**Figure 1:**
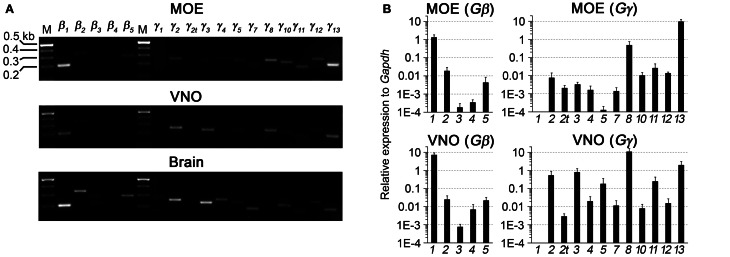
**RT-PCR and realtime qPCR analyses of Gβ and Gγ subunit gene transcript expression in sensory epithelia of the peripheral olfactory system. (A)** Results of two-step RT-PCR analysis, showing presence of multiple Gβ and Gγ subunit gene transcripts in these tissues. The PCR primers were designed against sequence fragments within 3′UTR of each member of the Gβ and Gγ subunit family. Total RNA was extracted from epithelium lining the olfactory turbinates (MOE) and sensory epithelium of the vomeronasal organ (VNO). Brain RNA extract was used as control. **(B)** Realtime qPCR analysis of Gβ and Gγ subunit gene transcript expression. The expression levels are plotted relative to *Gapdh* reference gene, showing strong expression of Gβ_1_, Gγ_8_, and Gγ_13_ gene transcripts in both epithelia and additional expression of Gγ_2_ and Gγ_3_ gene transcripts at lower levels in the VNO sensory epithelium.

**Figure A1:**
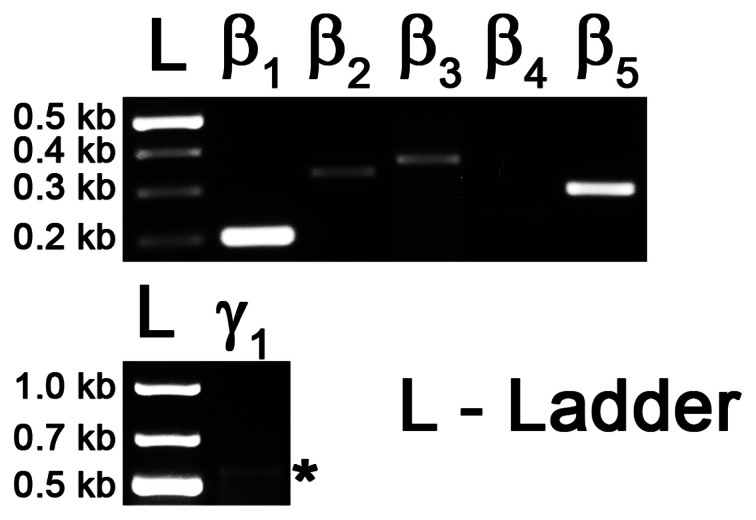
**RT-PCR on cDNA of eye tissue as positive control. Top:** Gel image showing the PCR products with primers against transcript sequences for Gβ_1_, Gβ_2_, Gβ_3_, Gβ_4_ and Gβ_5_. **Bottom:** Gel image showing PCR product of Gγ_1_ gene transcript. Asterisk ^*^ represents the expected size of the Gγ_1_ band (545 bp).

We first performed a coarse screening for the expression of G-protein βγ subunit gene transcripts in the main and accessory peripheral olfactory systems of mouse using RT-PCR. Gβ subunit members share 51–88% homology and Gγ members share 27–76% homology (Dupre et al., 2009). To maximize our chances of obtaining unique fragments for each Gβ and Gγ subunit, we generated oligonucleotide primers specific to the 3′ UTRs of each of the Gβ and Gγ gene transcript sequences to amplify regions that had lowest sequence homology (Table 1). Two-step RT-PCR was conducted using total RNA extracted from epithelium lining the mouse olfactory turbinates and vomeronasal sensory epithelium. Total RNA obtained from brain tissue was used as control. Figure 1A shows the RT-PCR results. In the MOE, we detected a strong band of amplicant product of Gβ_1_, moderate band of Gβ_2_, and weak bands of Gβ_4_ and Gβ_5_ gene transcripts. Of the 12 known Gγ subunits expressed in mammals, strong bands of amplicant products of Gγ_8_ and Gγ_13_ gene transcripts were observed in the MOE. We also observed moderate bands for Gγ_2_, Gγ_10_, Gγ_11_, Gγ_12_, and faint bands for Gγ_3_ and Gγ_5_ gene transcripts. In the VNO, RT-PCR results show a strong band of Gβ_1_ and a weak band corresponding to Gβ_2_ gene transcript. Among the Gγ subunits, we found strong bands for Gγ_2_, Gγ_3_, Gγ_8_, and Gγ_13_ amplicants and very faint band for Gγ_5_, Gγ_10_, Gγ_11_, and Gγ_12_ gene transcripts. For positive control, we used mRNA extracted from mouse brain (Figure 1A) and for some primers, such as Gγ_1_, we also used mRNA extracted from mouse eye tissue including the retina (Figure A1). The detected subunit sequence fragments were cloned into pGEM-T Easy vector and their identities were confirmed by sequencing. Thus our strategy of using primers to amplify specific 3′ UTR regions allowed us to obtain sequences highly specific to each Gβ and Gγ subunit. Our RT-PCR data suggests presence of multiple Gβ and Gγ subunit gene transcripts in the MOE and VNO.

### Quantitative analysis of Gβ and Gγ gene transcript expression in adult MOE and VNO

**Figure A2:**
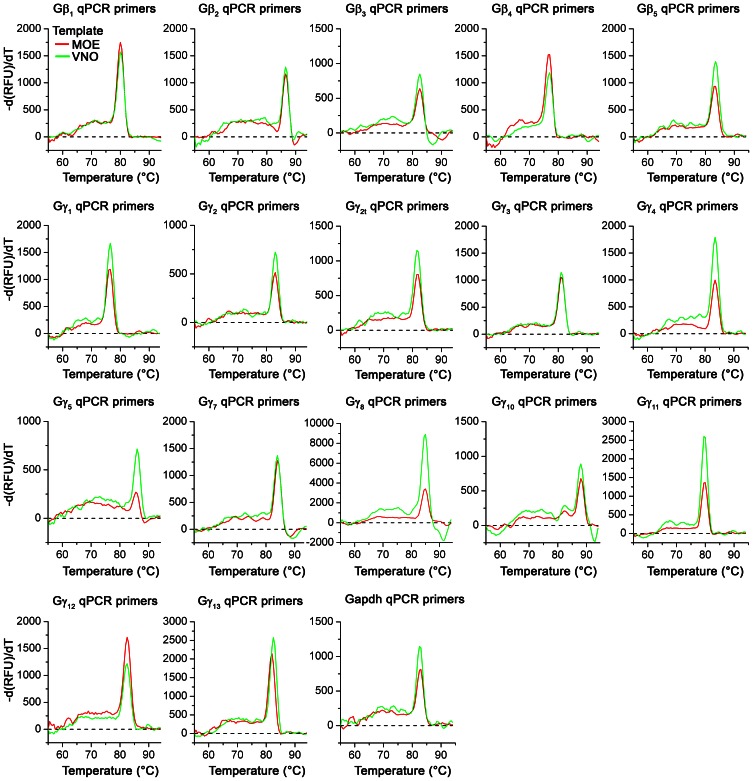
**Melting curves of realtime qPCR primer sets**. Representative melting curve data for all Gβ and Gγ gene transcripts using regular forward primer (Table 1, 5′) and qPCR reverse primers (Table 2, 3′) are shown. Melting curves were obtained as part of the regular qPCR protocol. Reactions with MOE and VNO cDNA as template are represented as red and green curves respectively. Note the presence of a single distinct peak in each curve for all primer sets with either MOE or VNO templates, demonstrating the primer specificity.

The significant variation in the band intensity of our RT-PCR results implicates different levels of Gβ and Gγ gene transcript expression. To obtain a quantitative measurement, we conducted qPCR to determine their expression relative to the glyceraldehyde 3-phosphate dehydrogenase (*Gapdh*) transcript. The specificity of the qPCR primers and products were determined by melting curve measurement, which show a single sharp peak for each subunit, indicating the primers and products are specific to our interest (Figure A2). In concordance with our RT-PCR results, qPCR on MOE extracts revealed a 1.3-fold higher relative expression for Gβ_1_ and a 0.5- and 9.3-fold higher expression for Gγ_8_ and Gγ_13_ gene transcripts respectively. Other Gβ and Gγ subunit gene transcripts were present in significantly lower quantities (One-Way ANOVA with Tukey's *post-hoc* analysis, *p* < 0.01, *n* = 3 mice) (Figure 1B, MOE). Similarly, in the VNO, we observed a 7-fold higher relative expression for Gβ_1_ and significantly lower expression for all other Gβ subunit gene transcripts (*p* < 0.01, *n* = 3 mice). Among Gγ subunits, we observed a 10.7-fold higher expression for Gγ_8_ (One-Way ANOVA with Tukey's *post-hoc* analysis, *p* < 0.01, *n* = 3 mice) and 0.5-, 0.8- and 1.9-fold higher expression for Gγ_2_, Gγ_3_, and Gγ_13_ gene transcripts, respectively (Figure 1B, VNO). These qPCR results support our initial finding from the RT-PCR experiments and further demonstrate differential expression among Gβ and Gγ gene transcripts with Gβ_1_ being the most dominant Gβ in both MOE and VNO and Gγ_13_ and Gγ_8_ the most dominant Gγ in the MOE and VNO, respectively.

### Determination of cell-type specific expression of Gβ subunit gene transcripts in the adult MOE using RISH

**Figure 2:**
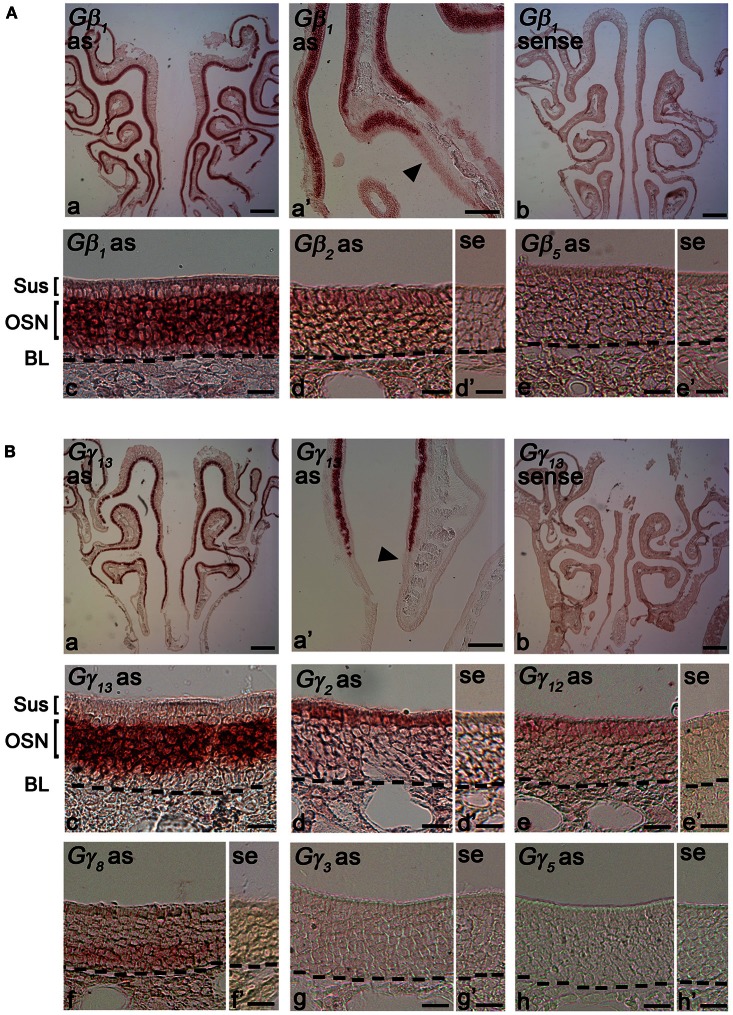
**RNA *in situ* hybridization analysis of Gβ and Gγ subunit gene transcript expression in the MOE. (A)** Gβ subunit gene transcripts expression in the MOE: (a) A low magnification image of a MOE coronal section showing Gβ_1_ gene transcript expression throughout the MOE. as: antisense probe. (a′) Gβ_1_ gene transcript expression is absent in the respiratory epithelium (arrowhead). (b) Gβ_1_ sense probe shows no labeling. (c) Higher-magnification image of Gβ_1_ gene transcript labeling in the MOE Sus: sustentacular/supporting cell layer, OSN: olfactory sensory neuron layer. BL: basal lamina—black dashed line. (d and d′) images of antisense and sense probe labeling of Gβ_2_ respectively, showing weak expression in supporting cell layer. se: sense probe. (e and e′) images of antisense and sense probe labeling of Gβ_5_, showing absence of Gβ_5_ gene transcript. **(B)** RISH analysis of Gγ subunit gene transcripts in the MOE: (a) A low magnification image of a MOE coronal section labeled with Gγ_13_ antisense probe, showing Gγ_13_ gene transcript expression throughout the MOE. (a′) Gγ_13_ gene transcript expression is absent in the adjacent respiratory epithelium (arrowhead). (b) Gγ_13_ sense probe labeling. No signal was observed. (c) A higher-magnification image of Gγ_13_ gene transcript labeling in the MOE. (d and d′) images of antisense and sense probe labeling of Gγ_2_, showing Gγ_2_ gene transcript expression in supporting cells. (e and e′) images of antisense and sense probe labeling of Gγ_12_. The Gγ_12_ gene transcript expression in supporting cells is weak. (f and f′) images of antisense and sense probe labeling of Gγ_8_, showing Gγ_8_ gene transcript expression in immature OSN region. (g and g′) images of antisense and sense probe labeling of Gγ_3_. (h and h′) images of antisense and sense probe labeling of Gγ_5_. Note there is no labeling for both Gγ_3_ and Gγ_5_ gene transcripts in the MOE. In **(A)** and **(B)**, scale for (a, b) is 0.2 mm, (a′) is 50 μm, (c, d, d′, e, e′) is 20 μm; In (B), scale for (f, f′, g, g′, h, h′) is 20 μm.

Gβ subunits have been shown to be involved in important cellular functions, such as chemotaxis and development (Zwaal et al., 1996; Peracino et al., 1998). In order to identify cell-type specific spatial patterns of Gβ subunit gene transcript expression in the peripheral olfactory sub-systems, we conducted RISH experiments on 14 μm-thick coronal nasal sections under high stringency conditions, with a hybridization temperature of 65°C for Gβ and Gγ transcripts detected through RT-PCR. We observed specific, strong labeling for Gβ_1_ gene transcript in the OSN layer throughout the entire MOE (Figure 2Aa). Our results are in agreement with an earlier study by Kerr et al. (2008) who also used RISH to identify signaling components that might partner with Ric8B, a guanine-nucleotide exchange factor (GEF) in olfactory neurons. Further, we found that Gβ_1_ gene transcript expression was fairly uniform throughout the MOE without any zonal bias, and is clearly absent in the respiratory epithelium (Figure 2Aa′). An image of the sense control is presented for comparison (Figure 2Ab). A relatively higher magnification image of Gβ_1_ gene transcript expression (Figure 2Ac) clearly shows that Gβ_1_ expression is restricted in OSNs and is not in supporting cell and basal stem cell layers. These results suggest an olfactory-specific role for Gβ_1_ in the nasal cavity.

Additionally, we found weak expression of Gβ_2_ gene transcript in the supporting cell layer (Figures 2Ad: antisense; d′: sense control). We did not observe any labels for Gβ_4_ and Gβ_5_ subunit gene transcripts in the MOE (Figures 2Ae: antisense; e′: sense control of Gβ_5_; Gβ_4_ data not shown), although positive signals of these two subunits were detected in RT-PCR (Figure 1A). We reasoned that high stringency in hybridization temperature (65°C) might affect the efficiency of RISH in detecting the weak expression of Gβ_4_ and Gβ_5_ gene transcripts, hence, we performed RISH with hybridization temperatures as low as 55°C. We did not find any increase in Gβ_4_ and Gβ_5_ labeling (data not shown). Thus, the expression of these two subunit gene transcripts, if present at all, is very weak.

### Cell-type specific expression of Gγ subunit gene transcripts in the adult MOE

Unlike the Gβ family, where most of its members share between 75–85% sequence homology to each other, Gγ subunits are more heterogeneous, sharing a broad range of homology in their amino acid sequences from 27% to 76% (Dupre et al., 2009). Such heterogeneity suggests that the Gγ subunits confer selectivity in signaling to the Gβγ dimer. We performed RISH on coronal nasal sections using antisense and sense probes generated against the seven Gγ subunit gene transcripts identified by our positive RT-PCR results. We found very strong expression of Gγ_13_ gene transcript in the olfactory epithelium throughout the MOE, voiding the respiratory epithelium (Figures 2Ba,a′, a sense control image in **b**). A relatively higher magnification image of Gγ_13_ expression is shown (Figure 2Bc). Clearly, Gγ_13_ gene transcript is expressed in the OSN layer only, which is consistent with previous findings showing the expression of Gγ_13_ protein in immunolabeling experiments (Lin et al., 2007; Li et al., 2013). In the supporting cell layer, we found moderate expression of Gγ_2_ (Figures 2Bd and d′: antisense and sense, respectively) and very weak expression of Gγ_12_ gene transcript (Figures 2Be and e′: antisense and sense, respectively). Additionally, we observed expression of Gγ_2_ gene transcript in the ventral respiratory epithelium (data not shown). Our RISH analysis for Gγ_3_ and Gγ_5_ gene transcripts, for which we found weak expression in our RT-PCR and qPCR, did not indicate significant expression in the MOE (Figures 2Bf and f′: antisense and sense for Gγ_3_. g and g′: antisense and sense for Gγ_5_). We conducted RISH experiments with antisense probes against Gγ_10_ and Gγ_11_. We observed very faint, ubiquitous expression for Gγ_11_ gene transcript in the OSN layer, and absence of label in the MOE for Gγ_10_ gene transcript (data not shown), thus, although these subunit gene transcripts may be present in the MOE, their quantity may be very insignificant. Finally, our RISH result shows Gγ_8_ gene transcript expression in the immature OSN population located in the lower portion of the OSN layer of the MOE, confirming a previous report by Ryba and Tirindelli (1995) (Figures 2Bf and f′: antisense and sense, respectively). Thus, our RISH analysis demonstrates distinct Gγ subunit gene transcript expression in different cell types of the MOE.

**Figure 3:**
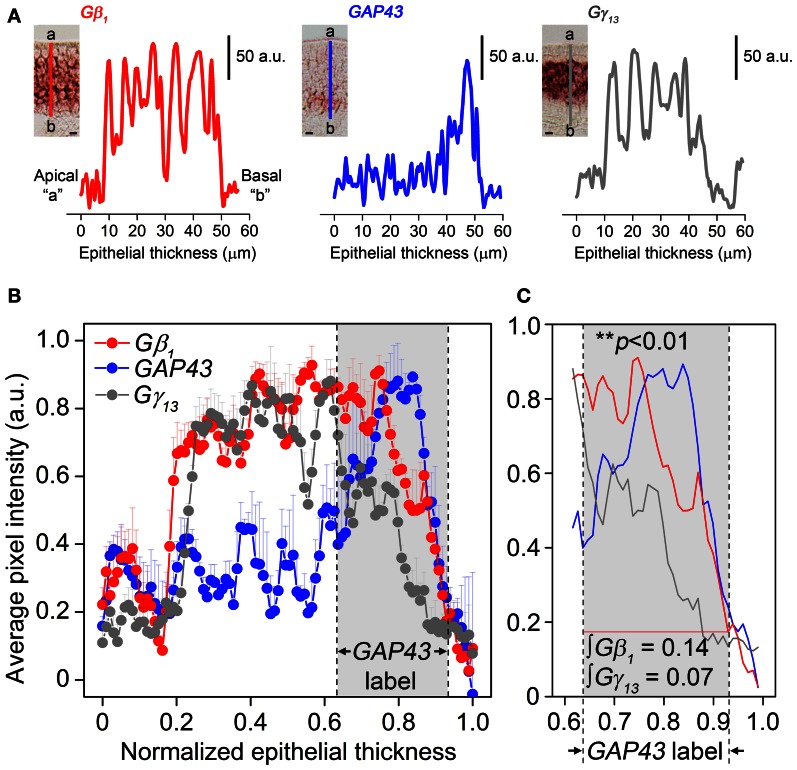
**Gβ_1_, GAP43, and Gγ_13_ gene transcript expression distribution in the MOE analysis using line intensity scan analysis. (A)** Representative line intensity scans of RISH signals of Gβ_1_, GAP43, and Gγ_13_ antisense probe labeling corresponding to line segments marked from “a” to “b” in insets. Insets: images of typical RISH labeling. The apical region is indicated as “a” and the basal lamina indicated as “b”. **(B)** Comparison of averaged line intensity scans for Gβ_1_ (red), Gγ_13_ (dark grey) and GAP43 (blue) (*n* = 3 mice per label). Error bars represent SEM on each point of averaged line scan of Gβ_1_ (red), Gγ_13_ (grey) and GAP43 (blue), respectively. Light-grey shaded box between dashed vertical lines corresponds to the region expressing GAP43. Strong, overlapping labels of Gβ_1_ and Gγ_13_ are observed in the upper portion of the OSN layer, where there is no label of GAP43 gene transcript. **(C)** Magnified view of the shaded region in **(B)**. Gγ_13_ signal drops shapely and disappears, whereas Gβ_1_ signal, although is reduced, remains and significantly overlaps with GAP43 label. “∫Gβ_1_” and “∫Gγ_13_” represent area integrals under Gβ_1_ (red line) and Gγ_13_ (grey line). The difference between these two distributions (over which area integrals are calculated) is statistically significant (Kolmogorov-Smirnov test, *p* < 0.01). Scale for insets in **(A)** is 10 μm.

Our results in Figure 2 also indicate that Gβ_1_ gene transcript expression pattern in the MOE is broader than that of Gγ_13_ gene transcript. Because only Gβ_1_ gene transcript is expressed in the OSN population, we hypothesized the Gβ_1_ gene transcript is expressed in both mature and immature OSNs. We performed RISH analysis for GAP43 gene transcript, an immature OSN marker and compared quantitatively the distribution pattern of GAP43, Gβ_1_, and Gγ_13_ gene transcripts in the MOE using line scan analysis (Sathyanesan et al., 2012). We measured various regions of the MOE and examples of the measurements from images of GAP43, Gβ_1_, and Gγ_13_ RISH are shown in Figure 3 (A: RISH images in inset; traces represent individual line scan denoted by the line segment from the apical region “a” to the basal lamina “b”—ab). We found that Gβ_1_ RISH signal distribution partially overlaps with that of GAP43 gene transcript in lower portion of the OSN layer, while in the upper 2/3 portion, it overlaps with Gγ_13_ RISH signal. In contrast, we found Gγ_13_ RISH signal distribution rarely overlaps with the distribution of GAP43 gene transcript signal (Figure 3C). Area integrals over the GAP43 gene transcript label (Figure 3, gray-shaded box in **B** and **C**) showed that the area covered by the Gγ_13_ trace is equal to only half the area covered by the Gβ_1_ trace (∫Gβ_1_ = 0.14, ∫Gγ_13_ = 0.07). A Kolmogorov-Smirnov test conducted between the Gβ_1_ and Gγ_13_ RISH signal distributions within the GAP43 RISH label revealed a significant difference between these distributions (*p* < 0.01). These results support our hypothesis that Gβ_1_ gene transcript is expressed in both immature and mature OSNs as well as confirm that Gγ_13_ gene transcript is restricted to mature OSNs.

**Figure 4:**
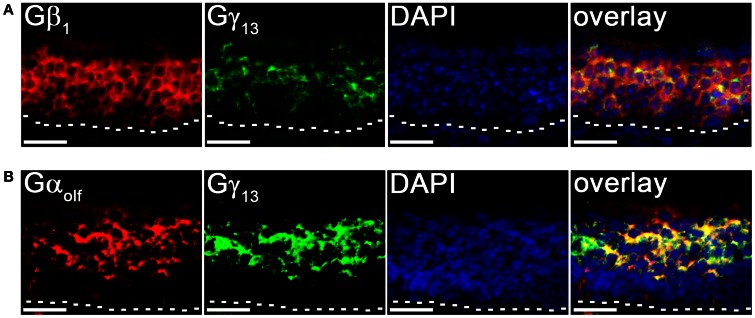
**Cell type-specific expression of Gβγ subunit gene transcripts in the MOE. (A)** Confocal images of MOE labeled with Gβ_1_ (red) and Gγ_13_ (green) fluorescent probes in double-probe RISH analysis. DAPI nuclear stain (blue). **(B)** Confocal images of double-probe RISH analysis in MOE sections using Gα_olf_ (red) and Gγ_13_ (green) fluorescent probes. DAPI nuclear stain (blue). White dashed lines represent basal lamina. Scale for all panels: 25 μm.

To further confirm our results of line scan analysis, we performed fluorescence double-label RISH. As shown in Figure 4A, the RISH labeling of Gγ_13_ is largely overlapped with Gβ_1_ on the upper OSN layer, where cell bodies of mature OSNs reside, while in lower portion of the OSN layer, there are more OSNs labeled with the Gβ_1_ probe than the Gγ_13_ probe. To confirm the line intensity scan result of the Gγ_13_ gene transcript express pattern, we performed double labeling for Gγ_13_ and Gα_olf_ gene transcript which is expressed in the mature OSNs and plays an essential role in odor transduction (Belluscio et al., 1998). We found their mRNA expression in largely the same population of OSNs (Figure 4B). These results suggest that Gβ_1_ most likely partners with Gγ_13_ in mature OSNs and in the immature OSNs population, Gβ_1_ partners with Gγ_8_.

### Expression of Gβ subunit gene transcripts in the VNO

**Figure 5:**
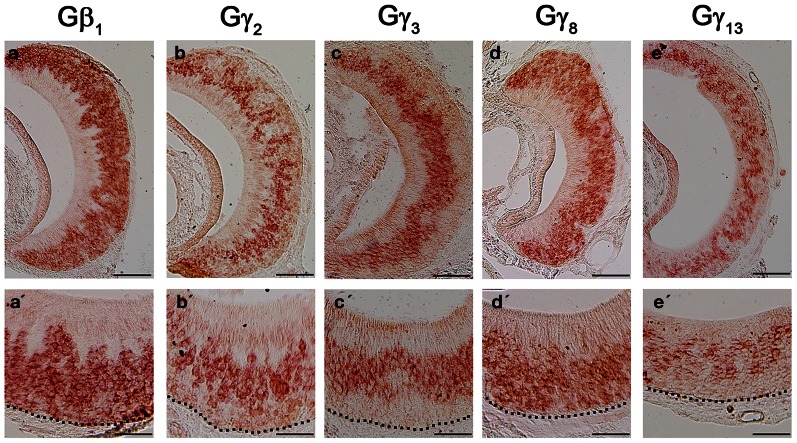
**RNA *in situ* hybridization analysis of Gβ and Gγ subunit gene transcript expression in the adult VNO. Top:** RISH images of coronal sections of VNO labeled with antisense probes against Gβ_1_ (a), Gγ_2_ (b), Gγ_3_ (c), Gγ_8_ (d) and Gγ_13_ (e); **Bottom:** Higher-magnification views of RISH images correlated to images in the top panels. Black dotted line represent basal lamina. Note that Gβ_1_ and Gγ_8_ are expressed throughout VSN layers, whereas Gγ_2_, Gγ_3_ and Gγ_13_ are expressed largely in the apical VSN zone. Scale: all top panels: 100 μm bottom panels: 50 μm.

Although the transduction pathway in VSNs is considered to be mediated through phospholipase C—a known target of Gβγ signaling, to our knowledge, no study so far has documented the systematic expression (or lack of expression) of Gβ or Gγ isotypes in the mouse VNO. We performed single-label RISH experiments to localize the expression of Gβ subunit gene transcripts in VNO coronal sections. We detected strong labeling for Gβ_1_ gene transcript in the VSN layer, including both apical and basal regions (Figures 5a,a′). We did not observe labeling for Gβ_1_ gene transcript in the supporting cell layer of the vomeronasal sensory epithelium, nor did we observe labeling in the convex non-sensory epithelium. This RISH result is consistent with our RT-PCR and qPCR data, strongly suggesting that Gβ_1_ is the dominant Gβ subunit gene transcript in the VSNs.

### Expression of Gγ subunit gene transcripts in the VNO

Among the subunit gene transcripts tested in our RISH analysis, we discovered expression of multiple Gγ subunit gene transcripts in VSNs including Gγ_2_, Gγ_3_, Gγ_8_, and Gγ_13_ (Figures 5b–e, with b′–e′ showing magnified images of the same subunits). Interestingly, all three subunit gene transcripts, Gγ_2_, Gγ_3_, and Gγ_13_ are expressed in the apical zone of the VSN layer, while Gγ_8_ is expressed throughout the entire VSN region. Our RISH result of Gγ_8_ is in agreement with an earlier study performed in rats, indicating the presence of Gγ_8_ in the vomeronasal sensory epithelium (Ryba and Tirindelli, 1995, 1997). However, the pattern f expression found in mice in our study was different from that seen in rats, in that we did not see increased labeling at the boundary between the sensory and non-sensory epithelia as Ryba and Tirindelli reported previously (Figures 5d,d′). These results from RISH analysis confirm our RT-PCR and qPCR results and further demonstrate heterogeneity in the expression of Gγ subunit gene transcripts.

**Figure 6:**
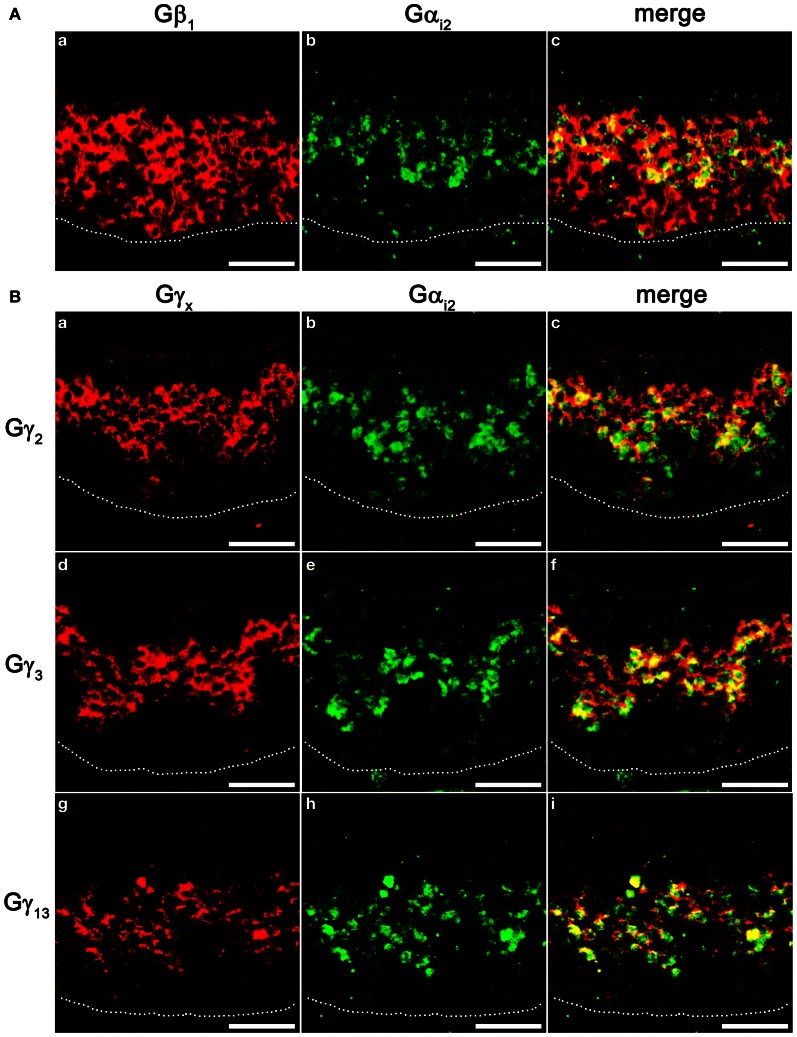
**Cell type-specific expression of Gβγ subunit gene transcripts in the VNO. (A)** Confocal images of VNO coronal sections labeled with Gβ_1_ (a, red) and Gα_i2_ (b, green) fluorescent probes in double *in situ* hybridization, respectively, and the overlay image (c). Gβ_1_ is expressed in both apical, Gα_i2_-expressing zone and basal zone lacking Gα_i2_. **(B)** Confocal images of VNO coronal sections double-labeled with antisense probes against Gγ_2_, Gγ_3_, Gγ_13_ (a, d, g, respectively, red) and Gα_i2_ (b, e, h, respectively, green) in *in situ* hybridization. The corresponding overlaid images are shown in c, f, i. Note that Gγ_2_, Gγ_3_, and Gγ_13_ are colocalized in the apical, Gα_i2_-expressing zone. Scale for all panels: 50 μm.

VSNs in the VNO are known to express two different Gα subunits in different zones. VSNs in the apical zone express Gα_i2_ while VSNs in the basal zone express Gα_o_. To further characterize the zonal specific expression of the Gβγ subunits in the VNO, we performed fluorescence double-label RISH using Gα_i2_ as a marker for the VSNs of the apical zone. Our results revealed that Gβ_1_ is expressed in both Gα_i2_-expressing apical VSNs and Gα_o_-expressing basal VSNs (Figures 6Aa–c). Thus, in the mouse VNO, Gβ_1_ most likely is the partner for both Gα_i2_ and Gα_o_ subunits. Double-label RISH analysis using probes against either Gγ_2_, Gγ_3_ or Gγ_13_ and Gα_i2_ revealed that these Gγ subunit gene transcripts are expressed in the apical zone of VSNs, voiding the basal zone (Figures 6Ba–i). Therefore, our results suggest the expression of multiple isotypes of Gγ subunit gene transcripts in the Gα_i2_-expressing population of VSNs and single Gβ and Gγ gene transcripts (Gβ_1_ and Gγ_8_, respectively) in the basal zone of Gα_o_-expressing VSNs. Table 3 summarizes all our results obtained from RT-PCR, qPCR and RISH.

**Table 3:** **Summary of RT-PCR, qPCR and RISH data on Gβγ expression**.

**Type**	**RT-PCR result (quality of band)**		**qPCR result (normalized to *Gapdh*)**		**RISH analysis**	
	**MOE**	**VNO**	**MOE**	**VNO**	**MOE**	**VNO**
**Gβ SUBUNITS**						
β_1_	Strong	Strong	1.31 ± 5.13E-01	7.05 ± 2.13	mOSNs and iOSNs	Gα_o_-VSNs and Gα_i2_-VSNs
β_2_	Very weak	Very weak	1.85E-02 ± 1.11E-02	2.44E-02 ± 1.50E-02	Sus (weak)	N.D.
β_3_	N.D.	N.D.	1.81E-04 ± 1.13E-04	7.58E-04 ± 3.15E-04	N.D.	N.D.
β_4_	Very weak	N.D.	3.39E-04 ± 1.38E-04	6.82E-03 ± 6.21E-03	N.D.	N.D.
β_5_	Weak	N.D.	4.24E-03 ± 4.23E-03	2.16E-02 ± 1.13E-02	N.D.	N.D.
**Gγ SUBUNITS**						
γ_1_	N.D.	N.D.	5.26E-06 ± 3.31E-06	1.11E-05 ± 7.13E-06	N.D.	N.D.
γ_2_	Weak	Strong	7.49E-03 ± 6.57E-03	5.31E-01 ± 3.51E-01	Sus (adult), basal cells (p7-p14)	Gα_i2_VSNs
γ_2*t*_	N.D.	N.D.	2.04E-03 ± 8.53E-04	2.87E-03 ± 1.18E-03	N.D.	N.D.
γ_3_	Very weak	Strong	3.24E-03 ± 1.10E-03	7.91E-01 ± 4.48E-01	N.D.	Gα_i2_-VSNs
γ_4_	N.D.	N.D.	1.60E-03 ± 1.06E-03	2.00E-02 ± 1.54E-02	N.T.	N.T.
γ_5_	Weak	Very weak	1.26E-04 ± 7.55E-05	1.80E-01 ± 1.78E-01	N.D.	N.D.
γ_7_	Very weak	N.D.	1.37E-03 ± 7.81E-04	1.13E-02 ± 9.98E-03	N.T.	N.T.
γ_8_	Moderate	Strong	4.75E-01 ± 2.71E-01	10.7 ± 5.53	iOSNs	Gα_o_-VSNs and Gα_i2_-VSNs
γ_10_	Weak	Very weak	1.01E-02 ± 4.64E-03	7.90E-03 ± 5.69E-03	N.D.	N.D.
γ_11_	Weak	Very weak	2.58E-02 ± 1.85E-02	2.46E-01 ± 1.85E-01	N.D.	N.D.
γ_12_	Weak	Very weak	1.34E-02 ± 2.32E-03	1.53E-02 ± 1.16E-02	Sus (weak)	N.D.
γ_13_	Strong	Strong	9.34 ± 3.38	1.95 ± 1.1	mOSNs	Gα_i2_-VSNs

qPCR results are quantities ± S.E.M., mOSN, mature OSN; iOSN, immature OSN; Sus, sustentacular cells; N.D., Not detected; N.T., Not tested.

### Expression patterns of Gβ_1_, Gγ_2_, Gγ_8_, and Gγ_13_ gene transcripts in early post-natal stages of the MOE

**Figure 7:**
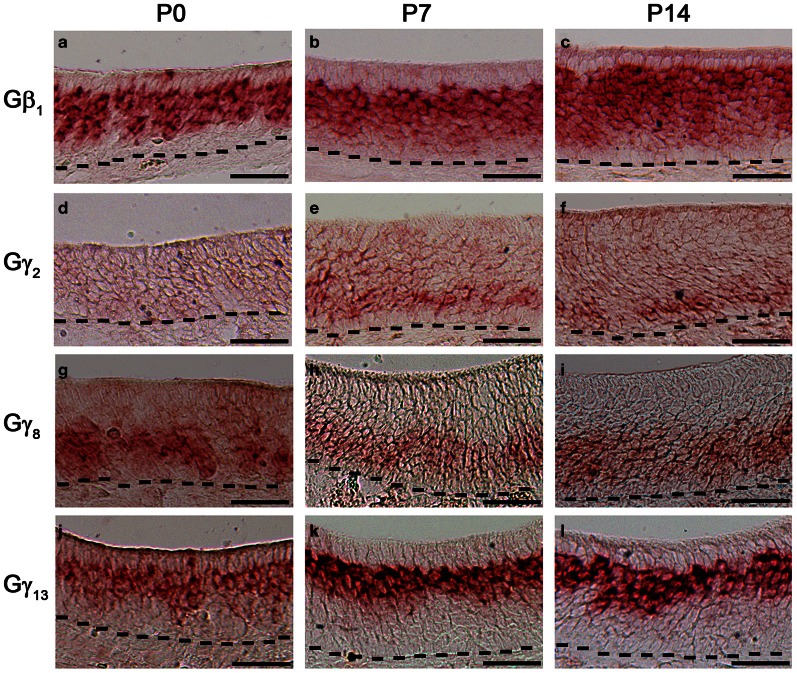
**Post-natal expression of Gβ_1_, Gγ_2_, Gγ_8_, and Gγ_13_ gene transcripts in the MOE**. RISH images of Gβ_1_, Gγ_2_, Gγ_8_, and Gγ_13_ antisense probe labeling at post-natal stages, P0 (a, d, g, j, respectively), P7 (b, e, h, k, respectively) and P14 (c, f, i, l, respectively). Note transient expression of Gγ_2_ at P7 and 14 and largely unchanged expression patterns of Gβ_1_, Gγ_8_, and Gγ_13_. Scale for all panels: 50 μm.

Changes in expression pattern of G-proteins in the MOE have been studied regarding Gα subunits. It has been established that as an OSN matures, its Gα expression profile shifts from expressing Gα_s_ during immature stage to expressing Gα_olf_ in its mature, functional stage (Imai et al., 2006; Chesler et al., 2007). Because many OSNs are mature at birth, Gα_olf_ expression can be found abundantly in the MOE in newborn animals (Sullivan et al., 1995). Post-natal expression analysis of the Gβ and Gγ subunit gene transcripts, however, has not been conducted for the mouse MOE. We extended our RISH screen of Gβ and Gγ subunit gene transcripts to three post-natal developmental timepoints—P0, P7, and P14. Our results indicate an increase in the number of layers of OSNs expressing Gβ_1_ gene transcript as the animals grow (Figures 7a–c). Gγ_2_ gene transcript expression was not present in P0 (Figure 7d). Unexpectedly, we observed a transient expression of Gγ_2_ gene transcript in a single layer of cells, which were ~1–2 cell-layers above the basal lamina at P7 and P14 (Figures 7e,f). The distribution patterns of Gγ_8_ and Gγ_13_ gene transcripts do not change postnatally with Gγ_8_ gene transcript located in the lower portion of the OSN layer (Figures 7g–i) while Gγ_13_ gene transcript is restricted in the apical region of the OSN layer (Figures 7j–l). Thus, except for the Gγ_2_ subunit, Gβ_1_, Gγ_8_, and Gγ_13_ gene transcripts closely retain their distribution patterns post-natally in the MOE.

### Expression patterns of Gβ_1_, Gγ_2_, Gγ_3_, Gγ_8_, and Gγ_13_ gene transcripts in early post-natal stages of the VNO

**Figure 8:**
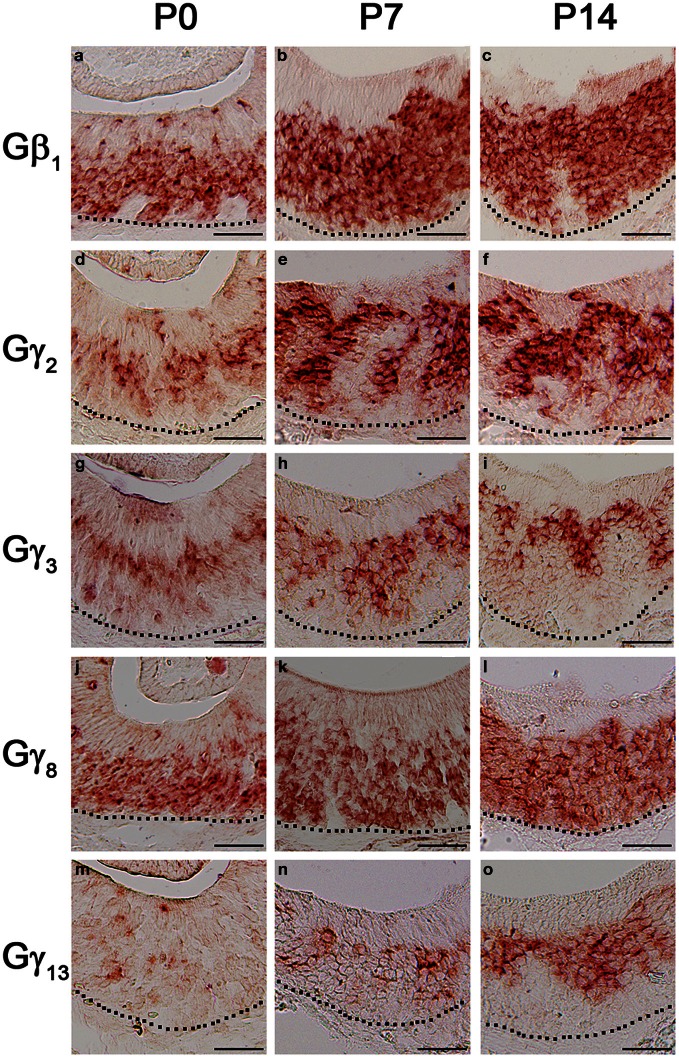
**Post-natal expression patterns of Gβ_1_, Gγ_2_, Gγ_8_, and Gγ_13_ gene transcripts in sensory epithelium of the VNO**. RISH images of Gβ_1_, Gγ_2_, Gγ_3_, Gγ_8_, and Gγ_13_ antisense probe labeling at post-natal stages, P0 (a, d, g, j, m, respectively), P7 (b, e, h, k, n, respectively) and P14 (c, f, i, l, o, respectively). The expression patterns are largely unchanged although the signals of Gβ_1_, Gγ_2_, Gγ_3_, and Gγ_13_ are increased postnatally. Scale for all panels: 50 μm.

The first 2 weeks after birth involve considerable cell proliferation in the VNO and is accompanied by the topographic targeting of the VSNs to specific regions in the accessory olfactory bulb (AOB) (Jia and Halpern, 1996; Weiler et al., 1999). Hence, we conducted our RISH analysis in P0, P7, and P14 mouse VNO sections. Our result does not show any major pattern changes in the Gβ_1_ gene transcript expression pattern although a greater number of cells are labeled as animals become older (Figures 8a–c). There are also no major changes in the expression patterns of the apically expressed Gγ_2_ and Gγ_3_ gene transcripts, although at P0, zonal restricted distributions are not as obvious as at P14 (Figures 8d–f and g–i, respectively). In addition, we occasionally observed, for these subunits, increased labeling in the non-sensory epithelium at P0, P7, and P14 than at adult, where we did not observe significant labeling (data not shown). Similar to Gβ_1_, Gγ_8_ gene transcript, which is expressed in both apical and basal VSNs, also did not show any major changes in expression pattern (Figures 8j–l). However, we observed increased Gγ_13_ gene transcript expression as post-natal development progresses (Figures 8m–o). Thus, our results suggest that Gβ_1_, Gγ_2_, Gγ_3_, and Gγ_8_ gene transcripts maintain their expression patterns throughout post-natal development and Gγ_13_ gene transcript expression increases as the animal reaches adulthood.

## Discussion

We have conducted a comprehensive analysis of Gβγ subunit gene transcript expression profile and their postnatal developmental occurrence in the murine peripheral olfactory system using RT-PCR, qPCR and RISH approaches. Our complementary results reveal a differential expression pattern of Gβ and Gγ subunits in the MOE. Whereas Gβ_1_ is expressed in both mature and immature OSNs, Gγ_13_ is expressed exclusively in mature OSNs, contrasting the expression of Gγ_8_ in the immature OSN layer. We also observed changes in the temporal expression patterns of Gγ_2_ which is transiently expressed between P7 and P14. In the adult VNO we observed differential labeling of Gβ_1_, Gγ_2_, Gγ_3_, Gγ_8_, and Gγ_13_. We found that Gβ_1_ and Gγ_8_ are expressed in both apical and basal zones of the VSNs, whereas Gγ_2_, Gγ_3_, and Gγ_13_ are expressed in apical zone only. To our knowledge, a systematic analysis of G-protein β and γ subunit gene transcript expression has not been conducted in the mouse MOE and VNO. Our analysis, therefore, enables a more complete understanding of the molecular components involved in the function and development of the mammalian peripheral olfactory system.

### Expression diversity of Gβγ subunits

Heterotrimeric G-proteins consist of α, β, and γ subunits. In most systems, while the α subunits have been studied extensively, the βγ subunits have been generally considered to play a more passive role of negatively regulating α subunit activation and returning it to its ground state (Dupre et al., 2009). However, many lines of evidence have strongly established the βγ dimer as having functional roles of their own, distinct from those associated with returning the α subunit to ground state. Differential expression of the 5 β and 12 γ subunits gene transcripts and proteins in particular mammalian cell types is an indication of specific Gβ and Gγ interactions and their unique functions. For example, in mammalian taste buds, G-protein α–gustducin is expressed in taste receptor cells that can be further divided based on the types of taste receptors expressed (Hoon et al., 1999). All these cells strongly express β_3_ and γ_13_ transcripts, while some also express β_1_ additionally. Because in these taste receptor cells, Gβγ plays a critical role in activating the PLC β_2_-TRPM5 signaling pathway mediating transduction of sweet, bitter, umami and fatty acid tastes (Perez et al., 2002; Zhang et al., 2007; Liu et al., 2011), addition of Gβ_1_, although not determined, might contribute to the coding strategy of these taste substances (Huang et al., 1999). Similarly, rod photoreceptors in the mouse visual system express β_1_, β_5_-l (β_5_-like, a splice variant of β_5_) and γ_1_. The Gα–transducin- β_1_- γ_1_ heterotrimer is involved in rhodopsin-mediated signal transduction (Kisselev and Gautam, 1993). Gβ_5_-l is found as a partner of RGS9, which is involved in signal termination (Keresztes et al., 2004; Nishiguchi et al., 2004). Thus, expression profiling of Gβγ subunits in a particular cell type is a helpful first-step in identifying specificities and generalities in signaling mechanisms.

The expression profile of Gβγ subunit gene transcripts in the adult mouse MOE as shown by our RT-PCR and qPCR results (Figure 1) suggests the expression of multiple β and γ subunits, albeit at different levels. While we observed strong RT-PCR and qPCR amplification of– β_1_, γ_8_, and γ_13_, which were reported previously (Ryba and Tirindelli, 1997; Lin et al., 2007; Kerr et al., 2008) we also noted weak or very weak levels of amplification of β_2_, β_4_, β_5_, γ_2_, γ_3_, γ_5_, γ_10_, γ_11_, and γ_12_ gene transcripts. A study similar to ours conducted in channel catfish olfactory neurons detected a smaller number of β (β_1_ and β_2_) and γ subunit gene transcripts (γ_2_ and γ_3_) (Bruch et al., 1997). The difference between our study and the study of Bruch et al. (1997) may be due to inherent differences in the olfactory systems of the two species. In addition, differences in tissue preparation may also contribute to the observed difference. Bruch et al. (1997) used cDNA from three dissociated olfactory neurons, whereas we extracted mRNA from peeled epithelium from olfactory turbinates. For all these subunits detected in MOE tissue, we also observed positive amplification from our control brain (Figure 1A) or eye tissue containing the retina (Figure A1) except β_4_, and γ_8_, suggesting that our primers are efficient and specific in amplifying specific subunit gene transcripts. Because these subunits are expressed in various brain regions and because we only took a small portion of the brain in our experiments, the lack of β_4_ product in the brain control most likely is due to the weak expression of this subunit (Ruiz-Velasco et al., 2002) and the small brain tissue we used rather than the efficiency of the primers.

In our study, RT-PCR expression profiling of Gβγ subunits in the VNO showed the presence of a single β subunit gene transcript, β_1_, and strong expression of four γ subunit gene transcripts, γ_2_, γ_3_, γ_8_, and γ_13_. Interestingly, Runnenburger et al. (2002) detected β_2_, γ_2_, γ_8_, and γ_11_ in rat VNO using PCR and immunohistochemistry (Runnenburger et al., 2002). Again, this difference could be species related (mouse vs. rat), however, different experimental approaches may also contribute to the variation. In fact, our results from RISH analysis do not completely agree with our RT-PCR (Table 3). In our study, the RT-PCR and qPCR data reflect expression in all four cells types composing the olfactory/vomeronasal epithelium, including supporting cells, OSNs, microvillar cells and progenitor cells. While data from RISH analysis allows discriminating between the various cell-types, the two complementary approaches do not have the same sensitivity. In fact, while we observed positive labeling in most of the subunits identified in RT-PCR screen, our RISH experiment failed to detect β_5_, γ_2_, and γ_5_ gene transcripts. In addition, because we extracted mRNA from peeled epithelium, our preparation might have been contaminated with small amount of blood cells, known to express various Gβγ subunits (Stephens et al., 1994; Neptune and Bourne, 1997; Welch et al., 2002; Lehmann et al., 2008).

### Cell type-specific expression of Gβγ gene transcripts in the MOE and VNO

Our RISH analysis of Gβγ gene transcript expression indicates striking similarities as well as differences between the MOE and VNO. Our data strongly demonstrate Gβ_1_ as being the dominant Gβ subunit expressed in both MOE and VNO sensory neurons which include mature and immature OSNs and VSNs, although in general the signal intensity in the mature neurons is stronger. No other Gβ subunit gene transcripts were detected from MOE or VNO sensory neurons in our RISH analysis, although light expression of Gβ_2_ gene transcript was seen in the supporting cell layer. This exclusive expression pattern is different from other sensory systems. For example, in mouse retina, Gβ_1_ is found enriched in retinal rod cells, whereas cone cells express both Gβ_1_ as well as Gβ_3_ (Huang et al., 2003). Differential expression of Gβ subunits is also seen among retinal bipolar cells; Rod bipolar cells express β_3_ and β_4_ and overlapping populations of cone bipolar cells express β_1_, β_2_, and β_5_ (Huang et al., 2003). Such differences may be due to differences in receptor-preference for particular G-protein combinations (Kleuss et al., 1993; Wang et al., 1997, 1999). Kerr et al. (2008) observed that Gβ_1_ was the dominant β subunit in the olfactory epithelium, however, considering the relatively widespread expression of Gβ_1_ (Malbon, 2005), it was unclear whether Gβ_1_ was exclusive to the neuroepithelium. In serial sections from anterior to posterior MOE, we noticed that Gβ_1_ gene transcript is exclusively expressed in the olfactory neuroepithelium and absent from the respiratory epithelium (Figure 2Aa′). Boto et al. (2010) also conducted a similar search for βγ subunits in *Drosophila* olfactory receptor organs. Although the authors of this study discovered the expression of two (β_5_ and β_13F_) out of the three known *Drosophila* β subunits and both the γ subunits (γ_1_ and γ_30A_) using RT-PCR, they only investigated the spatial expression of β_13F_. Our RISH results show that in the MOE, Gβ_1_, Gγ_2_, Gγ_8_, Gγ_12_, and Gγ_13_ gene transcripts are expressed in a cell-type specific manner with Gγ_2_ and Gγ_12_ gene transcripts being present only in supporting cells (Figure 2B). Based on the exclusive and strong expression of Gβ_1_ and Gγ_13_ and the colocalization of Gγ_13_ and Gα_olf_ gene transcripts in mature OSNs (Figure 3A), we speculate that Gβ_1_ and Gγ_13_ form a functional dimer of Gβ_1_−γ_13_ and serve as the cognate partner for Gα_olf_. This notion is in agreement with previous findings that Gβ_1_ and Gγ_13_ proteins are highly enriched cilia of the mature OSNs and that Gγ_13_ plays an important role in targeting other signaling proteins including Gα_olf_ to the cilia where olfactory transduction takes place (Kulaga et al., 2004; Lin et al., 2007; Kerr et al., 2008; Liu et al., 2012; Li et al., 2013). Because Gγ_13_ and Gα_olf_ gene transcripts are not expressed in the immature OSNs, Gβ_1_ may form a heterotrimeric G-protein complex with Gγ_8_ and Gα_s_, known to be present in these cells. Since our RISH data do not provide exact cellular location of the subunit proteins, further experiments are needed to confirm whether they indeed form a dimer and partner with Gα_s_ as well as to determine special functions of these dimers.

In the VNO, our results show a greater diversity in Gγ subunit gene transcript expression patterns. Surprisingly, the apical Gα_i2_-expressing VSNs express 4 different Gγ subunit gene transcripts—γ_2_, γ_3_, γ_8_, and γ_13_, while the basal Gα_o_-expressing VSNs express only Gγ_8_ gene transcript. We did not observe increased labeling of Gγ_8_ gene transcript at the boundary between the sensory and non-sensory epithelia, as reported by Ryba and Tirindelli (1997) in rat VNO. Based on our result of strong expression of Gβ_1_ gene transcript in both apical and basal VSNs, we speculate that different heterotrimers such as Gα_i2_- β_1_- γ_8_, Gα_i2_- β_1_- γ_2_, Gα_i2_- β_1_- γ_3_, Gα_i2_- β_1_- γ_13_ are present in apical VSNs *in vivo*, while in basal VSNs, however, Gα_o_- β_1_- γ_8_ likely is the only heterotrimer, unless hitherto undiscovered βγ subunits also exist. It is also possible that in apical VSNs, only one of the three Gγ subunit proteins may partner with Gα_i2_-Gβ_1_, even though three gene transcripts might be expressed. Future molecular and biochemical experiments are needed to determine their interactions and identify their cognate partners in the heterotrimeric complex in these VSNs.

### Developmental expression patterns of Gβγ in MOE and VNO

Knowledge about developmental regulation of βγ subunits in mammals is limited and in the olfactory system, this knowledge is missing (Norgren et al., 1995; Chesler et al., 2007). Yet, studies from other systems in invertebrate species, such as *Drosophila* and *C.elegans* have demonstrated important roles for Gβ and Gγ in development (Zwaal et al., 1996; Gotta and Ahringer, 2001; Schaefer et al., 2001; Yu et al., 2003; Izumi et al., 2004). Among mammals, Okae and Iwakura (2010) reported severe neural tube defects in mouse embryos deficient for Gβ_1_ resulting in large-scale lethality with none of the embryos surviving more than 2 days after birth (Okae and Iwakura, 2010). Mice in which Gβ_5_ had been genetically ablated had severe impairment in neural development of the cerebellum and the hippocampus resulting in deficiencies in motor learning and coordination (Zhang et al., 2011). These recent studies indicate that Gβ and by extension, Gγ subunits, play crucial roles in mammalian neurodevelopment. In our study of postnatal expression pattern of Gβγ subunits, we found largely unchanged expression pattern of the majority Gβγ subunits tested in both the MOE and VNO, suggesting their consistent function through postnatal development to adulthood.

## Conclusion

In summary, we have performed comprehensive analyses of Gβγ subunit gene transcript expression in the mouse MOE and VNO of adults and at various stages of postnatal development. Our results reveal expression of multiple Gβγ subunit gene transcripts in cell-type specific fashion, which is largely unchanged from postnatal stages to adulthood, suggesting their persistent functions in sensory neurons of the peripheral olfactory system.

### Conflict of interest statement

The authors declare that the research was conducted in the absence of any commercial or financial relationships that could be construed as a potential conflict of interest.
